# Biochemical Analysis of Pentraxin 3 and Fibrinogen Levels in Experimental Periodontitis Model

**DOI:** 10.1155/2012/809801

**Published:** 2012-08-15

**Authors:** Gonca Cayir Keles, Umut Balli, Burcu Ozkan Cetinkaya, Bulent Ayas, Arzu Findik, Zeynep Pinar Keles, Ferda Pamuk

**Affiliations:** ^1^Department of Periodontology, Faculty of Dentistry, Ondokuzmayis University, 55139 Samsun, Turkey; ^2^Department of Histology and Embryology, Faculty of Medicine, Ondokuzmayis University, 55139 Samsun, Turkey; ^3^Department of Microbiology, Faculty of Veterinary Medicine, Ondokuzmayis University, 55139 Samsun, Turkey; ^4^Department of Periodontology, Faculty of Dentistry, Istanbul Aydin University, 34380 Istanbul, Turkey

## Abstract

*Objective.* Pentraxin 3 (PTX3), newly discovered inflammation marker, is a member of acute-phase proteins. The hypothesis, synthesis of gingival tissue and serum PTX-3 increases in the experimental periodontitis model (with 10-day and 40-day periods), was tested by detecting gingival tissue and serum PTX-3 levels in rats with experimental periodontitis. *Methods.* Thirty rats were randomly divided into three groups of ten animals each: ligature-induced experimental periodontitis groups (with 10-day (Group1) and 40-day periods (Group2)) and healthy group (Group3). At the end of experimental period, rats were sacrificed, and radiological and histomorphometric analyses were performed on the mandibles. PTX3 levels were measured in gingival tissue and serum samples using ELISA. Plasma fibrinogen levels were measured according to the nephelometric method. *Results.* Significant alveolar bone resorption and periodontal inflammation were evident in periodontitis groups. Levels of PTX3 in gingival tissue were statistically higher in Group 1 than those in groups 2 and 3 (*P* < 0.01). No significant difference was found in serum PTX3 levels between experimental periodontitis and control groups (*P* > 0.05). Plasma fibrinogen levels were significantly increased in the experimental periodontitis groups (*P* < 0.001). *Conclusion.* PTX3 seems to be associated with tissue destruction in earlier periods of inflammatory periodontal disease, contrary to the fibrinogen findings.

## 1. Introduction

Periodontal disease is a multifactorial infectious disease; although the main cause of periodontal disease is the presence of periodontal microorganisms, subsequent progression and disease severity are considered to be determined by the host immune response [1–4]. Mediators produced as a part of host response that contribute to tissue destruction include acute-phase proteins, cytokines, and prostaglandins [5–8]. The acute-phase response is a nonspecific process that may occur in the initial host response to injuries, infections, ischaemic necrosis, or malignancy [9]. It is initiated by the activation of local macrophages and other cells (including fibroblasts and endothelial cells) and has a variety of functions including proinflammatory properties, activation of complement factors, neutralization of invasive pathogens, and stimulation of repair and regeneration of tissues [9, 10]. Data show that acute-phase proteins, plasma proteins, not only appear in acute inflammation, but also in longstanding, chronic conditions [10]. Acute-phase proteins are generally increased following a microbial infection [11]. It is, therefore, possible that acute-phase proteins are sensitive markers to evaluate inflammatory status of various microbial infections including periodontitis.

Pentraxins (PTX_S_), a superfamily of acute-phase proteins, are an essential component of the humoral arm of innate immunity [12, 13]. Short PTX_S_ such as C-reactive protein and serum amyloid P component are acute-phase proteins in man and mouse, respectively, and are produced mainly by the liver in response to inflammatory stimuli, such as IL-6 [14, 15]. PTX3 was the first long PTX described as an IL-1*β* inducible gene in endothelial cells [15]. It is produced by a variety of cells, mostly by cells abundant in periodontal tissues like neutrophils [8, 16], fibroblasts [8, 17], monocytes/macrophages [8, 17, 18], dendritic cells [8, 19], epithelial cells [8, 20], endothelial cells [8, 21], and vascular smooth muscle cells [8, 22]. PTX3 behaves as an acute-phase response protein since its blood levels, low in normal conditions (about 25 ng/mL in the mouse, <2 ng/mL in the man), increase rapidly (peak at 6–8 h) and dramatically (200–800 ng/mL) during endotoxic shock, sepsis, and other inflammatory and infectious conditions [23, 24].

There is evidence that PTX3 levels increase in various chronic inflammatory diseases such as atherosclerotic lesions [22, 24, 25], coronary artery disease [24, 26], small vessel vasculitis, rheumatoid arthritis [24, 27, 28], and chronic kidney disease [24, 29]. Moreover, in a recent clinical study, suggesting that PTX3 concentrations may have been a good predictive diagnostic tool for unstable angina pectoris, plasma, PTX3 levels have been significantly increased in patients with unstable angina pectoris [30]. To date, data suggest a possible role of PTX3 as a marker of pathology, since there is a correlation between PTX3 plasma concentrations and severity of diseases [24]. Periodontal disease is a low-grade local infection microorganisms and their products are the principal etiological agents, and it is associated with a moderate systemic inflammatory response [31, 32].

In light of these observations, PTX-3 might probably play a role in the pathogenesis of periodontal disease. The two recent clinical studies were published suggesting that PTX-3 in gingival crevicular fluid is considered a diagnostic marker of periodontal disease inflammatory activity [8, 33]. Fibrinogen, another acute-phase reactant, has been suggested to be a possible mediator in the pathogenesis of periodontal disease [34]. This is consistent with the report that there is an independent association between periodontal disease and plasma fibrinogen levels [35]. The present study was undertaken to test the hypothesis that synthesis of gingival tissue and serum PTX-3 increases in the experimental periodontitis model (with 10 days and 40 days periods) in rats which can easily be standardized. Plasma fibrinogen levels were also determined in rats with experimental periodontitis. 

## 2. Material and Methods

### 2.1. Animals

Thirty male Wistar rats weighing 200 to 250 g were housed separately in plastic cages and kept in a temperature controlled room with a standard light dark illumination cycle (12 hours each). They received water and standard food *ad libitum.* All study protocols were in compliance with guidelines and with the approval of the Committee of Ethics in Animal Research of the Ondokuzmayis University.

### 2.2. Experimental Design

Thirty rats were randomly divided into three groups of ten animals each: experimental periodontitis groups (with 10 days and 40 days periods) and periodontally healthy group. After systemic anesthesia with an intraperitoneal injection of 60 mg/kg ketamine-HCL (Pfizer, Istanbul, Turkey), the rats were subjected to experimental periodontitis by tying 3.0 sterile silk ligatures around the cervical area of the right and left mandibular first molars, and these were kept in position for 10 days (Group 1) and 40 days (Group 2) to promote microbial dental plaque accumulation, and inflammation [36]. At the end of the experimental period, 5 mL venous blood was drawn by cardiac puncture and processed for serum and plasma analyses. After that procedure, the rats with experimental periodontitis and periodontally healthy rats (Group 3) were decapitated. The mandibles were carefully removed together with the surrounding gingiva, and the gingival tissue samples were extracted from the buccal region of the mandibular right first molars. The left mandibles were soaked in neutral 10% formalin for 48 hours. Standardized radiographs were obtained by the long-cone technique at 70 kilovolt (peak), 8 mA from all groups.

### 2.3. Histomorphometric Analysis

Following the radiographs, the left mandibles were decalcified in 10% formic acid, embedded paraffin; 6 *μ*m thick sections were cut in a mesio-distal direction and stained with hematoxylin and eosin. The level of the alveolar bone was determined by histometric measuring the distance from the cementoenamel junction to the alveolar bone crest [36]. These measurements were performed on monitor images of the sections, which were transferred via color camera (objective ×3.3, F10 CCD Camera, Panasonic, Osaka, Japan) attached to a microscope (BH2 Research Microscope, Olympus, Tokyo, Japan). Inflammatory cells were counted in systematically sampled; 40 × 40-*μ*m areas.

### 2.4. Biochemical Analysis

Gingival tissues, removed from the alveolar bone, were placed immediately in a sterile saline solution and frozen at −80°C until biochemical analysis [37]. In brief, before grinding, tissue was blotted, weighed on a microbalance, and placed into a sufficient volume of phosphate buffered saline (PBS; 4°C; pH: 7.0) containing a protease inhibitor (5 *μ*g/mL aprotinin, 1 mM EDTA) to a dilution of 10 mg tissue/mL PBS plus protease inhibitor solution. The samples were homogenized at 8,500 revolutions per minute (rpm) for 30 seconds four times with 10-second intervals by homogenizer (Ultra Turrax. T25, IKA LABORTECHNIK, Staufen, Germany). The homogenate was processed with freeze-thawed procedures two times and then sonicated three times by ultrasonicator (MSE Soniprep 150, Sanyo Gallenkamp, Leicestershire, UK) at 4 to 5 *μ* for 30 seconds with 10-second intervals. The homogenate was centrifuged (Refrigerated centrifuge, SIGMA 3K30, Osterode, Germany) at 15,000 rpm for 16 minutes [37], and supernatant was collected for PTX3 analysis. The supernatant preparation processes were performed on ice medium at ~0°C to 4°C. All samples were brought to room temperature for enzyme-linked immunosorbent assay (ELISA). Gingival tissue and serum PTX3 concentrations were analyzed in each 50 *μ*L sample by standard ELISA apparatus at 450 to 550 nm using a PTX3 kit (Uscn Life Science Inc., Wuhan, China) that detects PTX3 levels. Plasma fibrinogen levels were measured according to the nephelometric method using a standard assay kit (Siemens Healthcare Diagnostics Products GmbH, Marburg, Germany).

### 2.5. Statistical Analysis

The statistical analysis was performed using a commercially available softcare program (SPSS 15.0; SPSS Inc., Chicago, IL, USA). Normalities of distributions were tested by Shapiro Wilk procedure. Mann-Whitney  *U*  nonparametric test was used for the intergroup comparisons of the data. The Spearman's Rank correlation test was also used to detect the relationship between biochemical and histomorphometric findings. Data are shown as means ± standard deviation and medians (minimum-maximum). Significant levels were calculated for  *P* < 0.05.

## 3. Results

**Figure 1:**
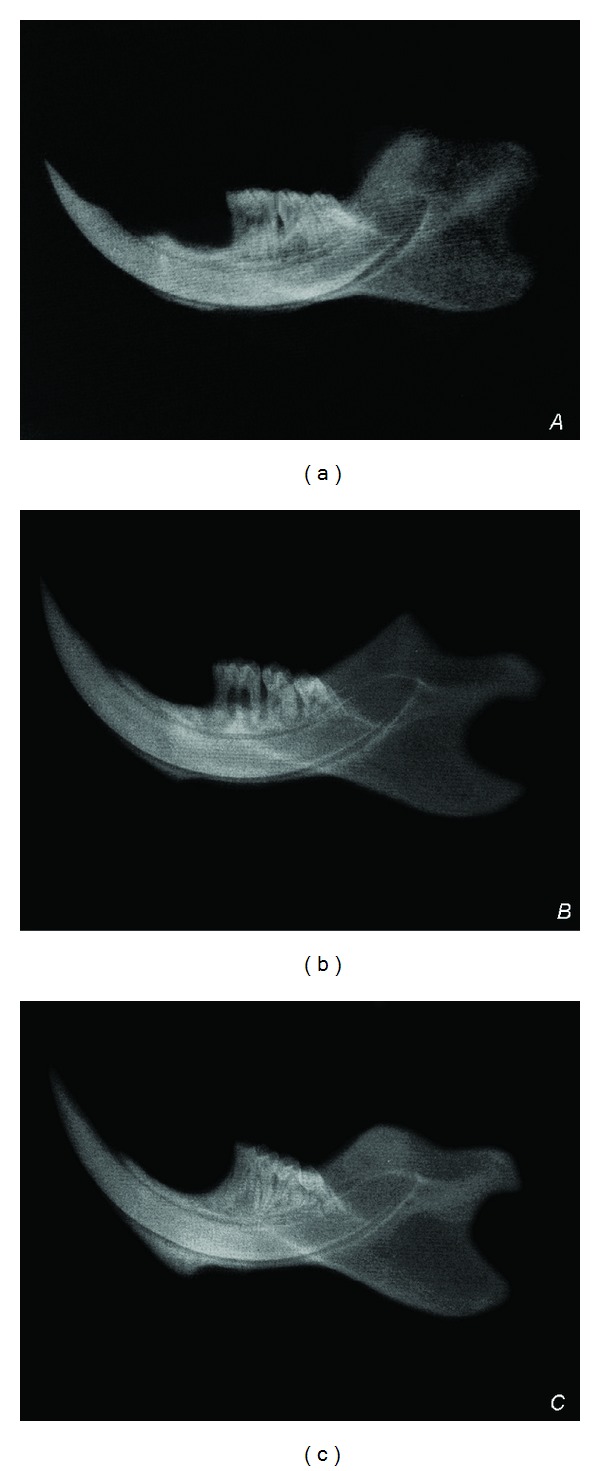
Radiological appearances of the periodontium. (a, b) Radiological alveolar bone resorption in the experimental periodontitis groups. (a) Group 1, (b) Group 2, and (c) radiologically healthy periodontium in the control group. (c) Group 3.

In the experimental periodontitis groups (Group 1 and Group 2), prominent alveolar bone resorption was observed on dental radiographs (Figures 1(a) and 1(b)). Radiologically healthy periodontium with no signs of alveolar bone loss was observed in the control group (Figure 1(c)).

### 3.1. Histomorphometric Findings

Histomorphometric findings are shown in Table 1.

**Figure 2:**
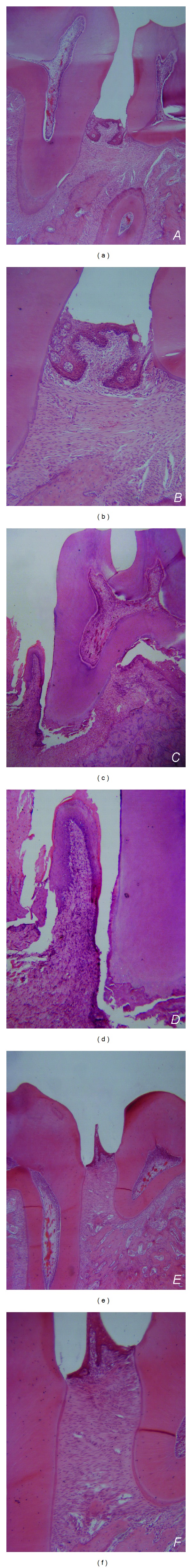
Photomicrographs of the periodontium from the mesial-distal sections of mandibular first molars. Significant alveolar bone resorption in Groups 1 and 2 (a–d), and healthy periodontium in Group 3 (e, f). Periodontal inflammation in experimental periodontitis groups (b, d). (a, b) Group1 (Magnification ×4, ×10), (c, d) Group 2 (Magnification ×4, ×10), and (e, f) Group 3 (Magnification ×4, ×10).

The distance from the cementoenamel junction to the alveolar bone crest both in Groups 1 and 2 was significantly higher than the control group (*P* < 0.001). There was also a significant difference between Groups 1 and 2 (*P* < 0.001). Significant alveolar bone resorption was detected in both periodontitis groups compared to the control group (Figure 2). Alveolar bone resorption was higher in Group 2 compared to Group 1.

The number of inflammatory cells both in the connective tissue and epithelium was significantly higher in Groups 1 and 2 compared to the periodontal healthy group (*P* < 0.001). There was also a significant difference between Groups 1 and 2 (*P* < 0.01). Significant periodontal inflammation was detected in both periodontitis groups (Figures 2(b), 2(d), and 2(f)).

### 3.2. Biochemical Findings

Findings of PTX3 are demonstrated in Table 2. Levels of PTX3 in gingival tissue were statistically higher in Group 1 than those in Groups 2 and 3 (*P* < 0.01). There was no significant difference between Groups 2 and 3 (*P* > 0.05). No significant difference was found in serum PTX3 levels between experimental periodontitis and control groups (*P* > 0.05).

Table 3 shows the plasma fibrinogen levels. Plasma fibrinogen levels were significantly increased in the experimental periodontitis groups (*P* < 0.001). There was also a significant difference between Groups 1 and 2 (*P* < 0.001). 

Correlation coefficients are shown in Table 4. A statistically significant positive correlation was found between levels of PTX3 in gingival tissue and alveolar bone level, levels of PTX3 in gingival tissue and number of inflammatory cells in epithelium, levels of PTX3 in serum and alveolar bone level, and also levels of PTX3 in serum and number of inflammatory cells in epithelium in Groups 1 and 2.

## 4. Discussion

In the present study, PTX3 levels were investigated in gingival tissue and serum of rats with experimental periodontitis. Experimental periodontitis characterized by infiltration of inflammatory cells and alveolar bone resorption was evident in the periodontal area both after 10 days and 40 days of ligature placement. The hypothesis tested is that synthesis of gingival tissue and serum PTX3 increases in the experimental periodontitis model (with 10 days and 40 days periods). Levels of PTX3 in gingival tissue were significantly higher in experimental periodontitis group after 10 days. In contrast to the hypothesis, there was no significant difference in gingival tissue PTX3 levels between experimental periodontitis after 40 days and periodontally healthy groups. The difference in the serum levels of PTX3 was not statistically significant between the study groups. The results of the present study clearly show that the concentration of PTX3 in gingival tissue and serum was positively correlated with alveolar bone resorption and with inflammatory cells in epithelium both in experimental periodontitis groups. Although the difference in the serum levels of PTX3 was not significant, there was a proportionate increase in serum levels from healthy controls to experimental periodontitis groups after 40 days to after 10 days. In the present study, the quantitative detections of PTX3 were performed by ELISA, which was generally used to detect PTX3 in gingival crevicular fluid and plasma with high sensitivity and specificity [8, 30, 33, 38].

The results on plasma fibrinogen levels in the present study confirm the role of this protein in the pathogenesis of periodontitis, which is higher in experimental periodontitis groups both after 10 days and 40 days. It is important to also consider that 40-day experimental period is sufficient for chronic periodontal inflammation in rats. This suggests that fibrinogen appears also in chronic inflammation process, which seems not to agree with PTX3. 

 The acute-phase reactant PTX3 is expressed as an IL-1 inducible and TNF inducible gene in endothelial cells and fibroblasts, respectively, [39–41]. PTX3 is mostly generated by endothelial cells and mononuclear phagocytes on stimulation with lipopolysaccharides and inflammatory cytokines, such as IL-1*β* and TNF-*α*, but not IL-6 [39, 41, 42]. CRP, IL-1*β*, IL-6, and TNF-*α* have been associated with the presence of various bacterial infections including periodontitis [9]. Periodontal disease is an inflammatory disease in which microorganisms and their products are the principal etiologic agents. In a recent clinical work, PTX3 is suggested to associate with the severity of periodontal disease and considered as a marker of inflammatory activity in periodontal disease [8]. It is important to note that there is no significant difference in plasma PTX3 concentrations between periodontal disease and control groups, which is in agreement with our serum PTX3 results. The results of the present study clearly showed that gingival tissue PTX3 levels were not increased in experimental periodontitis model with 40-day period, contrary to the gingival crevicular fluid findings of Pradeep et al. which reported that concentration of PTX3 in gingival crevicular fluid is increased in proportionately with the severity of periodontal disease [8]. Same authors in another clinical study observed that plasma PTX3 levels are higher in both patients with chronic kidney disease and with chronic kidney disease + periodontal disease than healthy controls. There is no significant difference in plasma PTX3 levels between two groups with chronic kidney disease [43]. Very recent clinical data suggested that gingival crevicular fluid PTX3 level is higher in periodontally diseased sites as compared to healthy sites in the same patient with chronic periodontitis [33]. Although the split mouth design is a good model in periodontal clinical studies, it is therefore possible that periodontopathogens in diseased sites may translocate to healthy sites. 

Periodontal tissue destruction occurs in an episodic, intermittent manner, with periods of inactivity and destructive periods that are associated with acute inflammatory reaction and result in loss of collagen and alveolar bone. It is important to also consider that the increased concentration of PTX3 in these clinical studies [8, 33] might probably be related to the destructive periods in periodontal disease. This is consistent with the results of the present study that levels of PTX3 in gingival tissue was significantly higher in experimental periodontitis group after 10 days. When our results were taken into consideration, a significant increase in the levels of PTX3 in gingival tissue occurred after 10 days of ligature placement due to the acute inflammatory reaction, contrary to the findings of experimental periodontitis group with 40-day period, which is chronic periodontal inflammation model.

 Some possible reasons for the inconsistent findings of previous clinical studies could be related to the characteristics of subjects. Existing knowledge demonstrated that physical activity and energy balance of individuals potentially influence inflammatory response [38]. Clinical data show that there is an inverse relationship between plasma levels of PTX3 and nutrient intake, and also body fat decreases. Moreover, the findings clearly indicate that bed rest increases the PTX3 plasma concentrations [38]. To overcome this problem, physical activity and diet of rats were standardized in the present study. Fibrinogen levels have been reported to be influenced by several factors such as age and gender [34]. From a clinician's perspective, it is notable that genetic predisposition, age, and gender can be standardized better in rats [33, 44].

## 5. Conclusion

The present study results clearly showed that PTX3 levels in gingival tissue were significantly higher in experimental periodontitis after 10 days but were not different from control at 40 days. To the best of our knowledge, this is the first study to investigate the relationship between PTX3 levels and experimental periodontitis with different stages. Within the limits of this study, it can be concluded that PTX3 seems to be associated with tissue destruction in earlier periods of inflammatory periodontal disease. 

## Figures and Tables

**Table 1:** Alveolar bone level and numbers of inflammatory cells counted in the connective tissue and epithelium.

	Alveolar bone level*	Inflammatory cells*	Inflammatory cells*
	(mm)	connective tissue	epithelium
		(cells/1600 *μ*m^2^)	(cells/1600 *μ*m^2^)
Group 1	1.08 ± 0.03	3.9 ± 0.7	3.4 ± 0.8
(Experimental periodontitis with 10 days period)	1.08 (1.03–1.13)	4.0 (3.0–5.0)	3.0 (2.0–5.0)
Group 2	1.37 ± 0.05	5.4 ± 1.2	4.5 ± 0.7
(Experimental periodontitis with 40 days period)	1.39 (1.29–1.44)	5.5 (4.0–7.0)	4.0 (4.0–6.0)
Group 3	0.93 ± 0.07	0.5 ± 0.5	0.3 ± 0.5
(Healthy control group)	0.96 (0.80–1.01)	0.5 (0.0–1.0)	0.0 (0.0–1.0)

Data were analyzed by one-way ANOVA and expressed as the means ± standard deviation and medians (minimum-maximum).

*Significant difference in histomorphometric findings between groups (*P* < 0.001).

**Table 2:** Gingival tissue and serum PTX3 levels in experimental periodontitis and control groups (ng/mL).

	Gingival tissue	Serum*
Group 1	1.6 ± 0.4^a^	1.2 ± 0.3
(Experimental periodontitis with 10 days period)	1.5 (1.3–2.4)	1.1 (0.9–1.6)
Group 2	1.2 ± 0.2^b^	1.1 ± 0.2
(Experimental periodontitis with 40 days period)	1.2 (1.0–1.5)	1.1 (0.9–1.4)
Group 3	1.1 ± 0.2^b^	1.0 ± 0.2
(Healthy control group)	1.1 (0.9–1.5)	1.0 (0.8–1.3)

Data were analyzed by one-way ANOVA and expressed as the means ± standard deviation and medians (minimum-maximum).

^a^Significant difference between groups 2 and 3 (*P* < 0.01).

^b^No significant difference between groups (*P* > 0.05).

*No significant difference in serum PTX3 levels between groups (*P* > 0.05).

**Table 3:** Plasma fibrinogen levels in experimental periodontitis and control groups (mg/mL).

	Plasma fibrinogen*
Group 1	1.73 ± 0.21
(Experimental periodontitis with 10 days period)	1.75 (1.36–2.10)
Group 2	0.99 ± 0.27
(Experimental periodontitis with 40 days period)	0.93 (0.74–1.69)
Group 3	0.67 ± 0.07
(Healthy control group)	0.72 (0.54–0.73)

Data were analyzed by Mann-Whitney *U* Test and expressed as the means ± standard deviation and medians (minimum-maximum).

*Significant difference in fibrinogen levels between groups (*P* < 0.001).

**Table 4:** Correlation coefficients between the levels of PTX3 in gingival tissue and serum, plasma fibrinogen levels, and histomorphometric findings in experimental periodontitis and control groups.

	GPTX3 to ABL	GPTX3 to ICE	GPTX3 to ICCT	STX3 to ABL	STX3 to ICE	STX3 to ICCT
Group 1	0.976	0.874	0.657	0.948	0.863	NA
	*P* < 0.001	*P* < 0.01	*P* < 0.05	*P* < 0.001	*P* < 0.01	
Group 2	0.982	0.908	NA	0.966	0.914	NA
	*P* < 0.001	*P* < 0.001		*P* < 0.001	*P* < 0.001	
Group 3	NA	0.646	NA	NA	NA	NA
		*P* < 0.05				

Data were analyzed Spearman's Rank Correlation Test.

GPTX3: levels of PTX3 in gingival tissue, SPTX3: levels of PTX3 in serum, ABL: alveolar bone level, ICE: inflammatory cells in epithelium, ICCT: inflammatory cells in connective tissue.

NA: not applicable.

## References

[1] Albandar JM, Brunelle JA, Kingman A (1999). Destructive periodontal disease in adults 30 years of age and older in the United States, 1988–1994. *Journal of Periodontology*.

[2] Renvert S, Lindahl C, Roos-Jansåker AM, Lessemi J (2009). Short-term effects of an anti- inflammatory treatment on clinical parameters and serum levels of c-reactive protein and proinflammatory cytokines in subjects with periodontitis. *Journal of Periodontology*.

[3] Honda T, Domon H, Okui T, Kajita K, Amanuma R, Yamazaki K (2006). Balance of inflammatory response in stable gingivitis and progressive periodontitis lesions. *Clinical and Experimental Immunology*.

[4] Keles GC, Cetinkaya BO, Eroglu C, Simsek SB, Kahraman H (2010). Vascular endothelial growth factor expression levels of gingiva in gingivitis and periodontitis patients with/without diabetes mellitus. *Inflammation Research*.

[5] Loos BG, Craandijk J, Hoek FJ, Wertheim-Van Dillen PME, Van Der Velden U (2000). Elevation of systemic markers related to cardiovascular diseases in the peripheral blood of periodontitis patients. *Journal of Periodontology*.

[6] Slade GD, Offenbacher S, Beck JD, Heiss G, Pankow JS (2000). Acute-phase inflammatory response to periodontal disease in the US population. *Journal of Dental Research*.

[7] De Nardin E (2001). The role of inflammatory and immunological mediators in periodontitis and cardiovascular disease. *Annals of Periodontology*.

[8] Pradeep AR, Kathariya R, Raghavendra NM, Sharma A (2011). Levels of pentraxin-3 in gingival crevicular fluid and plasma in periodontal health and disease. *Journal of Periodontology*.

[9] Ide M, McPartlin D, Coward PY, Crook M, Lumb P, Wilson RF (2003). Effect of treatment of chronic periodontitis on levels of serum markers of acute-phase inflammatory and vascular responses. *Journal of Clinical Periodontology*.

[10] Loos BG (2005). Systemic markers of inflammation in periodontitis. *Journal of Periodontology*.

[11] Lydyard PM, Cole MF, Lamont RJ, Burne RA, Lantz MS, Leblanc DJ (2006). The immune system and host defense. *Oral Microbiology and Immunology*.

[12] Pepys MB, Baltz ML (1983). Acute phase proteins with special reference to C-reactive protein and related proteins (pentaxins) and serum amyloid A protein. *Advances in Immunology*.

[13] Norata GD, Marchesi P, Pulakazhi Venu VK (2009). Deficiency of the long pentraxin ptx3 promotes vascular inflammation and atherosclerosis. *Circulation*.

[14] Garlanda C, Bottazzi B, Bastone A, Mantovani A (2005). Pentraxins at the crossroads between innate immunity, inflammation, matrix deposition, and female fertility. *Annual Review of Immunology*.

[15] Maina V, Cotena A, Doni A (2009). Coregulation in human leukocytes of the long pentraxin PTX3 and TSG-6. *Journal of Leukocyte Biology*.

[16] Jaillon S, Peri G, Delneste Y (2007). The humoral pattern recognition receptor PTX3 is stored in neutrophil granules and localizes in extracellular traps. *Journal of Experimental Medicine*.

[17] Goodman AR, Levy DE, Reis LFL, Vilcek J (2000). Differential regulation of TSG-14 expression in murine fibroblasts and peritoneal macrophages. *Journal of Leukocyte Biology*.

[18] Alles VV, Bottazzi B, Peri G, Golay J, Introna M, Mantovani A (1994). Inducible expression of PTX3, a new member of the pentraxin family, in human mononuclear phagocytes. *Blood*.

[19] Doni A, Michela M, Bottazzi B (2006). Regulation of PTX3, a key component of humoral innate immunity in human dendritic cells: stimulation by IL-10 and inhibition by IFN-*γ*. *Journal of Leukocyte Biology*.

[20] Nauta AJ, De Haij S, Bottazzi B (2005). Human renal epithelial cells produce the long pentraxin PTX3. *Kidney International*.

[21] Gustin C, Delaive E, Dieu M, Calay D, Raes M (2008). Upregulation of pentraxin-3 in human endothelial cells after lysophosphatidic acid exposure. *Arteriosclerosis, Thrombosis, and Vascular Biology*.

[22] Klouche M, Peri G, Knabbe C (2004). Modified atherogenic lipoproteins induce expression of pentraxin-3 by human vascular smooth muscle cells. *Atherosclerosis*.

[23] Dias AAM, Goodman AR, Dos Santos JL (2001). TSG-14 transgenic mice have improved survival to endotoxemia and to CLP-induced sepsis. *Journal of Leukocyte Biology*.

[24] Mantovani A, Garlanda C, Doni A, Bottazzi B (2008). Pentraxins in innate immunity: from C-reactive protein to the long pentraxin PTX3. *Journal of Clinical Immunology*.

[25] Rolph MS, Zimmer S, Bottazzi B, Garlanda C, Mantovani A, Hansson GK (2002). Production of the long pentraxin PTX3 in advanced atherosclerotic plaques. *Arteriosclerosis, Thrombosis, and Vascular Biology*.

[26] Kotooka N, Inoue T, Fujimatsu D (2008). Pentraxin3 is a novel marker for stent-induced inflammation and neointimal thickening. *Atherosclerosis*.

[27] Luchetti MM, Piccinini G, Mantovani A (2000). Expression and production of the long pentraxin PTX3 in rheumatoid arthritis (RA). *Clinical and Experimental Immunology*.

[28] Fazzini F, Peri G, Doni A (2001). PTX3 in small-vessel vasculitides: an independent indicator of disease activity produced at sites of inflammation. *Arthritis and Rheumatism*.

[29] Tong M, Carrero JJ, Qureshi AR (2007). Plasma pentraxin 3 in patients with chronic kidney disease: associations with renal function, protein-energy wasting, cardiovascular disease, and mortality. *Clinical Journal of the American Society of Nephrology*.

[30] Inoue K, Sugiyama A, Reid PC (2007). Establishment of a high sensitivity plasma assay for human pentraxin3 as a marker for unstable angina pectoris. *Arteriosclerosis, Thrombosis, and Vascular Biology*.

[31] Listgarten MA (1987). Nature of periodontal diseases: pathogenic mechanisms. *Journal of Periodontal Research*.

[32] Keleş GÇ, Çetinkaya BÖ, Şimşek SB, Köprülü D, Kahraman H (2007). The role of periodontal disease on acute phase proteins in patients with coronary heart disease and diabetes. *Turkish Journal of Medical Sciences*.

[33] Fujita Y, Ito H, Sekino S, Numabe Y (2012). Correlations between pentraxin 3 or cytokine levels in gingival crevicular fluid and clinical parameters of chronic periodontitis. *Odontology*.

[34] Sahingur SE, Sharma A, Genco RJ, De Nardin E (2003). Association of increased levels of fibrinogen ad the -455G/A fibrinogen gene polymorphism with chronic periodontitis. *Journal of Periodontology*.

[35] Schwahn C, Völzke H, Robinson DM (2004). Periodontal disease, but not edentulism, is independently associated with increased plasma fibrinogen levels. Results from a population-based study. *Thrombosis and Haemostasis*.

[36] Keles GC, Acikgoz G, Ayas B, Sakallioglu E, Firatli E (2005). Determination of systemically & locally induced periodontal defects in rats. *Indian Journal of Medical Research*.

[37] Cetinkaya BO, Acikgoz G, Ayas B, Aliyev E, Sakallioglu EE (2006). Increased expression of vascular endothelial growth factor in cyclosporin A-induced gingival overgrowth in rats. *Journal of Periodontology*.

[38] Bosutti A, Malaponte G, Zanetti M (2008). Calorie restriction modulates inactivity-induced changes in the inflammatory markers C-reactive protein and pentraxin-3. *Journal of Clinical Endocrinology and Metabolism*.

[39] Breviario F, D’Aniello EM, Golay J (1992). Interleukin-1-inducible genes in endothelial cells. Cloning of a new gene related to C-reactive protein and serum amyloid P component. *Journal of Biological Chemistry*.

[40] Lee GW, Goodman AR, Lee TH, Vilcek J (1994). Relationship of TSG-14 protein to the pentraxin family of major acute phase proteins. *Journal of Immunology*.

[41] Napoleone E, Di Santo A, Bastone A (2002). Long pentraxin PTX3 upregulates tissue factor expression in human endothelial cells: a novel link between vascular inflammation and clotting activation. *Arteriosclerosis, Thrombosis, and Vascular Biology*.

[42] Alles VV, Bottazzi B, Peri G, Golay J, Introna M, Mantovani A (1994). Inducible expression of PTX3, a new member of the pentraxin family, in human mononuclear phagocytes. *Blood*.

[43] Pradeep AR, Kathariya R, Arjun Raju P, Sushma Rani R, Sharma A, Raghavendra NM (2011). Risk factors for chronic kidney diseases may include periodontal diseases, as estimated by the correlations of plasma pentraxin-3 levels: a case-control study. *International Urology and Nephrology*.

[44] Nishikawa S, Nagata T, Morisaki I, Oka T, Ishida H (1996). Pathogenesis of drug-induced gingival overgrowth. a review of studies in the rat model. *Journal of Periodontology*.

